# A typology of organizational readiness for change based on a latent profile analysis

**DOI:** 10.3389/fpsyg.2024.1453836

**Published:** 2024-12-23

**Authors:** Alina Köhler, Marie Ritter, Simone Kauffeld

**Affiliations:** Department of Industrial/Organizational and Social Psychology, Institute of Psychology, Technical University Braunschweig, Braunschweig, Germany

**Keywords:** organizational readiness for change, change management, typology, antecedents, latent profile analysis

## Abstract

Companies have to undergo many change processes to succeed in the transforming economy. However, many change processes fail because employees are insufficiently accompanied through the process in a targeted manner. This study of N = 427 employees from a steel industry company undergoing a transformation process examines whether the organizational readiness for change (ORC) of highly affected employees can be classified into profiles, how these profiles can be predicted by various antecedents, and whether outcome variables such as job satisfaction can be explained by profile membership. Based on five facets of ORC (i.e., individual valence and positive affect), a total of six ORC profiles were identified: *Proactives*, *Acceptors*, *Opens*, *Neutrals*, *Reluctants* and *Deniers*. Employees’ optimism and the degree of perceived interpersonal and informational fairness can predict profile membership. It was shown that profiles significantly differ in relevant outcome variables satisfaction and intention to leave. These results contribute to the basic understanding of ORC and provide an initial approach for improving ORC profiles which could increase the success rate of change processes in companies.

## 1 Introduction

The ability of companies to adapt to change is crucial in a modern and rapidly changing work environment which is characterized by high uncertainty and complexity. Companies need to adjust to new circumstances, recognize new opportunities and minimize risks (Gaubinger, 2021; Stutz et al., 2021). Change processes occur, for example, in the context of digitalization, decarbonization, demographic change, or decentralization in organizations (Armenakis and Harris, 2009; Bennett and Lemoine, 2014; Bickenbach et al., 2022). A critical factor for the success of change processes in companies is the organizational readiness for change (ORC) of individual employees, as this forms the basis for motivated, effective work in times of change (Armenakis et al., 2007; Klonek and Kauffeld, 2012; Logan and Ganster, 2007). Therefore, understanding ORC and developing targeted intervention strategies to promote employee ORC is of great importance (Weiner, 2009). This paper aims to provide a new typology of ORC in organizational context that incorporates cognitive, affective, and behavioral aspects and that can serve as a basis for strategies promoting employee ORC during change processes.

ORC is seen as the tendency of individuals or groups to engage with and actively support or initiate forthcoming changes. In this study, the individual perspective is focused. The multidimensional construct ORC includes affective, cognitive and behavioral components (Gräfe and Kauffeld, 2023; Piderit, 2000). The cognitive component refers to individual beliefs about change, while the affective component describes the emotional reaction to change (Piderit, 2000). The behavioral component includes actual engagement in change processes and the willingness to engage in behaviors that support the change (Weiner, 2009). This tripartite division offers the advantage that employees can be assessed separately on all dimensions, and that intrapsychic ambivalences can be reflected. This is especially important, considering that employees might know how relevant the change is for the company and themselves (cognition) but still have negative emotions toward the change (e.g., Blanchette and Richards, 2010).

Gräfe and Kauffeld (2023) identified a 5-factor structure in which organizational valence, individual valence, positive affect, negative affect, and change behavior constitute the overarching factor of ORC. In this context, organizational valence is described as the perceived necessity and appropriateness of organizational changes and the associated benefits, while individual valence is the extent to which employees expect benefits from the change process for themselves individually. Affective appraisals of the change process are described by the extent of positive and negative affect, respectively. Lastly, change behavior describes change-related behaviors, e. g. seeking information and supporting colleagues (Gräfe and Kauffeld, 2023). This 5-factor structure can provide a better understanding of the diversity of responses to change. This can be essential in the identification of potential barriers and promotive factors for change as well as the development of targeted measures to overcome them (Holt et al., 2007).

**Figure 1:**
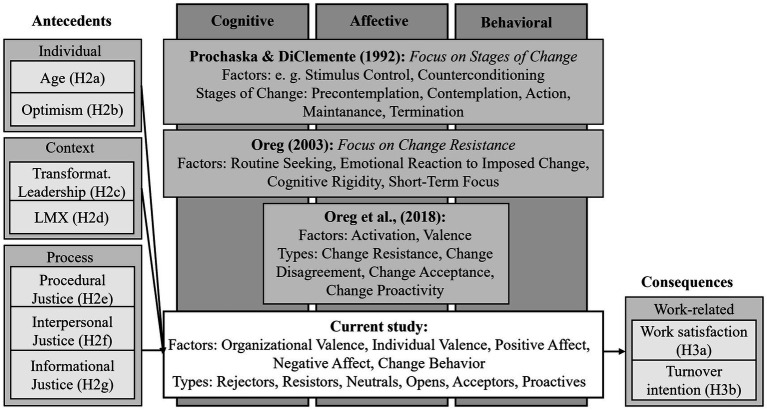
Framework of former studies in context of ORC typologies and current study.

Some works have attempted to identify patterns within the reactions to change. An overview of the different models can be seen in Figure 1. First, the Transtheoretical Model of Change by Prochaska and DiClemente (1992) conceptualizes change as a processual event in various stages, from pre-contemplation to maintenance. This model emphasizes the development of ORC at the individual level but does not fully capture the specific emotional and cognitive processes underpinning these stages and how individual and organizational factors interact to promote ORC. Second, Oreg (2003) developed a typology to measure individual differences in resistance for change, focusing on the factors Routine Seeking, Emotional Reaction to Imposed Change, Cognitive Rigidity, and Short-Term Focus. This theory provides an important contribution to understanding individual predispositions toward change but focuses primarily on the tendency to resist. Additionally, this work mainly focuses on individual characteristics that influence ORC instead of illuminating how individual reactions to change processes occur. Third, the Circumplex Model of Recipients’ Reactions to Change highlights the affective aspects of ORC by identifying various behavioral reactions to change based on the dimensions of activation and valence (Oreg et al., 2018). This model extends the understanding of the affective component of change readiness but neglects the cognitive and behavioral aspects of ORC. Here, only affect is used as the basis for the typology, assuming that behavior is related to it. However, Oreg et al. (2018) themselves note that emotion and behavior are not always consistent (e.g., Jordan et al., 2002; Martin et al., 1998). Since Gräfe and Kauffeld (2023) show, however, that affect as well as cognition and behavior form the overall ORC, this research gap should be urgently closed. The described models all share the assumption that individuals react differently to change. They differ, e.g., in the factors that define these reactions and whether the focus is on ORC or change resistance. Another distinction is whether they assume phases of change where individuals develop through the process, or they define fixed classes (Oreg et al., 2018; Prochaska and DiClemente, 1992). Prochaska and DiClemente (1992) developed a model focused on Stages of Change, whereas Oreg et al. (2018) created fixed classes, and Oreg (2003) emphasized Change Resistance. The models also vary in context: The Transtheoretical Model was originally developed for clinical psychology and later transferred to organizational context. The other models directly target the organizational context (Prochaska and DiClemente, 1992; Klonek et al., 2005; Oreg, 2003; Oreg et al., 2018). The models described form the basis of this study, as their different foci should be combined in this study.

In addition to existing theories about how people react to change, there is a lot of research into what factors ORC is related to (e.g., Holt et al., 2007; Oreg et al., 2011). In order to have an opportunity to increase employees’ ORC, it is relevant for companies to know which factors are related to the construct. So, to understand what makes an employee open to chance, the factors that influence ORC should be known. Holt et al. (2007) emphasize that ORC is influenced by individual attributes, process, context and content variables. Individual attributes include personal characteristics and experiences of employees (e.g., demographics, traits). Process variables refer to the specific way change processes are communicated, implemented, and supported by the organization (e. g. participation, justice). Context factors encompass the organizational and external circumstances under which the change takes place (e. g. trust, job characteristics). Content factors refer to what is being changed and are not considered in this study as the content of the change (steel production is being transitioned to a more sustainable production with less CO2 emissions) and its´ communication is equal for all employees surveyed in this study (Holt et al., 2007). Oreg et al. (2011) confirmed the 4-factor model and further integrated change consequences, which they divided into work-related (e.g., job satisfaction, turnover intention) and personal consequences (e.g., health, well-being). The model shows that the 4 factors of antecedents influence the explicit reaction to the change, which they divide into affective (e.g., stress, pleasantness), cognitive (e.g., change evaluation, change beliefs) and behavioral reactions (e.g., behavioral intentions, coping behaviors). They also describe an influence of the explicit reaction on several consequences (Oreg et al., 2011).

In addition to the described models, further studies measured antecedents and consequences of ORC: Previous research shows, for example, a negative correlation between age which can be considered as an individual attribute and ORC (Madden et al., 2010; Kunze et al., 2013). This result suggests that younger employees are generally more willing to accept changes than older employees. This insight is supported by further research showing that the duration of employment, often correlated with age, is positively associated with resistance to changes (Iverson, 1996; Van Dam et al., 2008). The individual attribute optimism, defined as the general expectation that more good things will happen in the future than bad, optimismis a key element of positive psychological capital (Scheier et al., 2021). Psychological capital is negatively associated with individual cynicism toward changes (Avey et al., 2011; Luthans and Youssef-Morgan, 2007). A significant aspect of the relationship between optimism and ORC lies in how optimistic individuals perceive and cope with challenges and stressors associated with change processes. Optimism promotes the development of coping strategies focused on problem-solving and positive reevaluation, facilitating adaptation to changes and enhancing resilience against potential negative impacts of these changes (Goel and Wani, 2024; Scheier et al., 1994). Moreover, optimism is associated with the ability to build and utilize a supportive social network, which is an important resource during organizational changes. These social support mechanisms can not only help reduce the perceived stress of changes but also positively influence the overall attitude toward change (Luthans et al., 2007). Transformational leadership is a context factor of ORC and can be characterized by idealized influence, inspirational motivation, intellectual stimulation, and individual consideration (Bass and Riggio, 2006; Saad Alessa, 2021). Through transformational leadership, employees experience more positive emotions regarding the change process, show less resistance and cynicism, and more ORC (Soe and Oreg, 2012; Bommer et al., 2005; Herold et al., 2008). Another context variable is Leader Member Exchange (LMX). High LMX quality is characterized, among other things, by strong trust, high support, and interaction between leaders and their employees (Graen and Uhl-Bien, 1995; Martin et al., 2018). Studies show that LMX is negatively associated with resistance to change and that it has an impact on appropriateness of the change, need for change and understanding of the change (Van Dam et al., 2008; Banguntopo, 2018). Furthermore, justice is considered a context variable and can be divided into procedural, interpersonal, informational, and distributive justice (Colquitt, 2001; Hadi et al., 2020; Bobocel, 2021). Procedural justice refers to the perception of fairness of the measures used in implementation and decision-making (Colquitt, 2001; Taylor, 2003). Interpersonal justice refers to the perceived appropriateness in social interactions, and informational justice refers to the perceived fairness of whether information is communicated truthfully, specifically, and timely (Maier et al., 2007; Kyei-Poku, 2019; Gim and Ramayah, 2019). Distributive justice pertains to the fair distribution of resources. It is assumed that there are no differences in distributive justice within the considered sample since the company’s co-determination ensures that employees’ needs are strongly represented and that the interests of employees are taken into account, thus ensuring a fair distribution of resources (Schwering, 2010). Therefore, this aspect is not focused on in this study. Generally, organizational justice perceived by employees positively correlates with individual commitment to change and negatively with cynicism (Armenakis et al., 2007; Bernerth et al., 2007). One study shows that procedural as well as interpersonal and informational justice significantly relate to all five factors of ORC (Gräfe and Kauffeld, 2023). Besides antecedents, further studies show that ORC correlates positively with the acceptance of change and negatively with turnover intention which can be considered as consequences of ORC (Wanberg and Banas, 2000; Oreg et al., 2011). Further, ORC can lead to, e.g., higher corporate behavior, commitment to change, job involvement and effort as well as to better teamwork and performance (Coyle-Shapiro and Morrow, 2003; Lok et al., 2005; Morgeson et al., 2006; Mossholder et al., 2000; Olafsen et al., 2021; Weiner, 2009).

## 2 Current research

This paper aims to gain a deeper, holistic understanding of the diverse reactions to organizational change while simplifying the complexity of ORC to make it practical for use. The understanding of individual reactions to change builds the basis of encouraging ORC and so to improve the results of change (Oreg et al., 2011). The development of ORC profiles serves this purpose by distilling individual reactions to change into categories that integrate the various facets of ORC. Without such typologizing, patterns are difficult to identify, making it challenging to compare reactions across individuals. Typologies are essential tools for simplifying organizational complexities, allowing for a better understanding of dynamic relationships and their impacts (Fiss, 2011). In practice, an ORC typology is highly relevant for organizations, as individualized measures to increase ORC for each employee are often impractical. Therefore, determining profiles and providing type-specific interventions can help companies design strategic, targeted change communication. The profiles in this study are developed through Latent Profile Analysis (LPA), which creates subgroups that differ in cognition, affect, and behavior which are the three constructs on which the profiles are based (Spurk et al., 2020). While previous studies have identified patterns of employee ORC, none have simultaneously integrated all three facets, despite the strong recommendation from researchers (Rafferty et al., 2013). For example, Oreg et al. (2018) created a typology based solely on affect, which they criticized themselves, as affect alone does not adequately explain cognition and behavior (Cooke and Sheeran, 2004; Trafimow et al., 2004). Moreover, many existing models neglect the integration of both personal and organizational valence. This model fills a critical gap by aligning personal motivations with organizational goals, which is essential for ensuring long-term success in dynamic business environments. The ORC-Q, which considers ORC multidimensionally within a five-factor structure, is the only tool specifically designed for the organizational change context and is thus used as the basis for this study (Gräfe and Kauffeld, 2023). This model aligns personal motivations with organizational goals, which is crucial as described above. Nonetheless, the variety of existing models highlights different patterns of reactions to change, underlining the importance of further exploration in this area (Oreg, 2003; Oreg et al., 2018; Prochaska and DiClemente, 1992).

It is a common fact, that “typically researchers have only a limited basis for predicting the nature and number of groups” (Marsh et al., 2009, p. 204–205). Further, meta-analysis from Spurk et al. (2020) showed, that 60.9% of reviewed articles conducting LPA’s (in context of vocational behavior) did not have hypotheses on number or shape of profiles which makes in common practice to have a general hypothesis. In this study, specific patterns in the five factors of ORC-Q cannot be predicted due to the lack of existing literature that provides clear, empirically supported models for patterns involving cognition, affect, and behavior. Instead, this study adopts an exploratory approach. It is conceivable that different types will exhibit varying levels of change behavior, analogous to the behavioral intention stages in the Transtheoretical Model (Prochaska and DiClemente, 1992). It is also possible to find different levels of affect, as seen in Oreg et al. (2018) model. However, it remains unclear how cognition and behavior might be shaped by varying levels of affect. For instance, it is possible that these dimensions are more pronounced in a type with strong positive affect compared to types with less positive affect. Another possibility is that there could be, e.g., a strongly affective type with low levels of cognition and behavior. In summary, since no specific patterns can be theoretically predicted, the study broadly assumes different types of ORC can be identified (Hypothesis 1). An overview of the hypotheses can be found in Figure 1.

When developing a typology, predictors of profile membership can be identified, offering additional insights to both research and practice. However, previously developed typologies could not provide insights in this regard (Oreg, 2003; Oreg et al., 2018; Prochaska and DiClemente, 1992). In In this study, we use the 4-factor model from Holt et al. (2007) as a basis for this. The factor content is not taken into account, as the sample is subject to the same change process (change to a low CO2 steel making). Since from the company’s perspective only a limited amount of data could be collected, preliminary discussions about the surveyed variables were held with the company which were based on practical, theoretical and data protection aspects. So, to two three variables per factor were chosen which are relevant in a theoretical and practical manner. The individual attributes selected were age and optimism, while the contextual variables were transformational leadership and LMX. For the process variables, three facets of justice were selected: specifically, procedural, interpersonal, and informational justice (Holt et al., 2007; Oreg et al., 2011). In context of consequences, the focus is deliberately placed on the work-related consequences of ORC, as these are directly connected to processes and dynamics within organizations (Harter et al., 2009; Griffeth et al., 2000). While personal-related consequences are also of interest, they have been intentionally excluded because they do not impact the organizational context to the same extent and are therefore less relevant for this research (Harter et al., 2009; Griffeth et al., 2000). In the discussion with the company about the consequences, the variables turnover intention and job satisfaction were selected as the key consequences to be considered. Since, as described, no specific types can be predicted due to a lack of theoretical literature, we cannot name concrete profiles in the following hypotheses. Nevertheless, hypotheses without referring to specific characteristics of profiles can often be found in previous research (e.g., Maynard et al., 2012; Rettew et al., 2008). We predict that younger employees (Hypothesis 2a), employees with high levels of optimism (Hypothesis 2b), employees who report high values of transformational leadership (Hypothesis 2c) and LMX (Hypothesis 2d) are more likely to be assigned to the profile with the highest ORC than to the profile with the lowest ORC. Moreover, it is expected that employees who perceive high procedural (Hypothesis 2e), interpersonal (Hypothesis 2f), and informational (Hypothesis 2 g) justice regarding the change process are more likely to belong to the class with the highest ORC than to the class with the lowest ORC. Those predictors can be practical starting points for influencing ORC profiles, specifically. For example, we can predict that people with certain characteristics are most likely to be assigned to a certain ORC type. Lastly, typologies enable type-specific predictions on company-relevant outcome variables (Fiss, 2011). It is expected that individuals belonging to the profile with the highest ORC have higher job satisfaction (Hypothesis 3a) and a lower intention to leave (Hypothesis 3b) than individuals belonging to the profile with the lowest ORC. So, the results of this study enable long-term predictions about, for example, the intention to leave of the various types in a change process, without having to capture the intention to leave separately. This represents a significant benefit for companies, saving time and financial resources. In sum, using a typology approach to ORC combined with antecedents and consequences allows to investigate complex cause-and-effect relationships in the context of ORC in reduced complexity while still considering various individual reactions (Fiss, 2011).

## 3 Materials and methods

### 3.1 Procedure and participants

Data collection took place between May and July 2022. In total, 427 employees of a German production company participated in a paper-pencil survey so that a total of around 34% of the employees that were particularly affected took part in the survey. All participants in the study are currently undergoing a transformation process, wherein steel production is being transitioned to a more sustainable production with less CO2 emissions. This process, in turn, has significant implications for the respondents’ jobs and tasks. The transformation process is accompanied by, e.g., the loss of traditional activities and jobs as well as new sustainable technologies (Köhler and Kauffeld, 2024). The survey was conducted on a voluntary basis and was subsequently distributed to all volunteers at information events about the change. It can be assumed that the people who took the questionnaire and spent the time to fill it out were more likely to be open to the change and want to stay with the company for longer. The employees were 40 years old on average. The sample was judged to be representative in terms of age as, e.g., the number of employees older than 60 years was 10% in the whole departments and 11% in this study’s sample. The gender distribution was unbalanced due to a men-dominated industry which is also representative for the surveyed company sector. So, 98% of participating employees were male, 1% female and 1% diverse. 42% of participants were leaders and 48% had no leadership position. All in all, the sample size is considered to be representative.

### 3.2 Measures

In this study, original German items in the measurements described below were used. These originals as well as additional information can be found in the Appendix.

*ORC* was measured by the Organizational Readiness for Change Questionnaire (ORC-Q; Gräfe and Kauffeld, 2023, *α* = 0.69). The ORC-Q consists of the three scales with a total of 15 items: One scale is valence, which has has two subscales, organizational valence (α = 0.86) and individual valence (α = 0.86). The second scale is affect, which also has two subscales positive affect (α = 0.94) and negative affect (α = 0.87). The third scale is change behavior (α = 0.73). Each subscale consists of three items scaled from 1 (= “do not agree at all”) to 5 (= “fully agree”). German items were used in the study. A translated example item of the organizational valance scale is “The Change is important for our company.”

*Optimism* was measured by the SOP2 (Kemper et al., 2013, *ω* = 0.94). It consists of two items, one concerning optimism (ω = 0.79) and one recorded item concerning pessimism (ω = 0.60). In the study, we used the original German items which can be translated to “Optimists are people who look to the future with confidence and usually expect good things. How optimistic are you in general?” and “Pessimists are people who look to the future with confidence and usually expect good things. How pessimistic are you in general?.” The scale is from 1 (= not at all) to 7 (= very much).

*Tranformational leadership* was measured by the German validated version of the MLQ-5 x Short based on MLQ Multifactor Leadership Questionnaire by Bass and Avolio (1995) (Felfe, 2006, *α* = 0.97). Through 20 items, the dimensions idealized influence, individualized consideration, intellectual stimulation, and inspirational motivation are measured and refer to the direct leader of the participants in this study (Felfe, 2006). The scale is from 1 (= never) to 5 (= almost always). An example item from Bass and Avolio (1995) is “Talks optimistically about the future.”

*Leader member exchange* was captured by a German leader member exchange scale based on Graen and Uhl-Bien (1995) 7-item scale (Schyns, 2002). The German version also includes seven items on a scale ranging from 1 (= never/not at all/not at all true) to 5 (= always/very good/highly true/totally true). An example item from Graen and Uhl-Bien (1995) questionnaire where the German version is based on is “How well does your leader understand your job problems and needs?”

*Organizational justice* including distributive, procedural and interpersonal justice was mapped by the German validated version of a questionnaire by Colquitt (2001) which’s overall scale consists of 20 items (Maier et al., 2007). We used the scales procedural justice (α = 0.86), interpersonal justice (α = 0.85) and informational justice (α = 0.91; Maier et al., 2007; Streicher et al., 2008, α = 0.94). As explained earlier, the distributive justice is not focused on this study. The range of values runs from 1 (= not at all/almost never) to 5 (= fully/often). An example item of the procedural justice scale from the questionnaire of Colquitt (2001) where the used scale is based on is “Have you been able to express your views and feelings during the change process?.” An example item of the interpersonal justice scale is “Has (he/she) treated you in a polite manner?” (Colquitt, 2001). An example item from the informational justice scale is “Has (he/she) communicated details in a timely manner?” (Colquitt, 2001).

*Job satisfaction* was measured using a modified version of the Job Diagnostic Survey based on Hackman and Oldham (1975) (Kil et al., 2000, α = 0.80) which consists of seven items. The value range extends from 1 (= strongly disagree) to 5 (= strongly agree). An example item from Hackman and Oldham (1975) is “Generally speaking, I am very satisfied with this job.”

*Intention to leave* was measured by the questionnaire on the turnover intention by Baillod and Semmer (1994, α = 0.80) which consists of four items and runs on a scale from 1 (= very rarely or small or very unlikely) to 5 (= very often or very large or very likely or very unlikely). A translated example item is “How often do you think about leaving your job?”

### 3.3 Statistical analyses

Statistical analyses were conducted in MPlus software (Muthén and Muthén, 2012). To test Hypotheses 1 a latent profile analysis (LPA) was calculated. An LPA is a classification method that aims to identify person-oriented superordinate classes based on specific variables and is based on a statistical model that shows the group membership modeled as a categorical latent variable (Ferguson et al., 2020). To calculate group membership, the means of ORC-Q-scales were used here (Gräfe and Kauffeld, 2023). The prerequisites were an entropy of ≤0.75 and that the loglikelihood can be replicated. Several models were calculated and the best model was selected based on the statistical criteria Akaike information criterion (AIC), Bayesian information criterion (BIC), sample size adjusted BIC (SABIC) and Entropy.

To analyze how age, optimism, transformational leadership, LMX, procedural, interpersonal, and informational justice relate to ORC profiles, a multinomial logistic regression was performed using the R3STEP command in MPlus (Asparouhov and Muthén, 2014). This analysis tests how an increase in the predictor is related to a higher probability of belonging to a specific ORC profile. To determine the relationship of ORC profiles on the outcome variables job satisfaction and intention to leave, the DU3STEP command in MPlus was used, predicting whether the mean values of the outcome variables differ between ORC profiles (Asparouhov and Muthén, 2014). Predictors and distal outcome variables were analyzed separately (Lanza et al., 2013). Subsequent post-hoc tests with Bonferroni correction were conducted to analyze which groups differed specifically in terms of distal outcomes.

## 4 Results

Table 1 shows descriptive statistics as well as manifest product–moment correlations of scales and scale reliabilities.

**Table 1:** Item characteristics and product–moment correlations of scales.

	N	M	SD	(1)	(2)	(3)	(4)	(5)	(6)	(7)	(8)	(9)	(10)	(11)	(12)	(13)	(14)
(1) Organ. valence	427	4.37	0.77	(0.71)													
(2) Ind. valence	424	2.92	0.99	0.29**	(0.94)												
(3) Positive affect	426	3.36	1.03	0.56**	0.58**	(0.95)											
(4) Negative Affect	424	2.59	0.95	−0.33**	−0.22**	−0.47**	(0.90)										
(5) Change Behavior	425	3.27	0.89	0.25**	0.27**	0.35**	−0.04	(0.76)									
(6) Age	418	40.94	10.80	0.04	0.76	0.03	−0.06	0.13**	–								
(7) Optimism	418	5.30	1.28	0.19**	0.24**	0.34**	−0.19**	0.18**	0.09	(0.71)							
(8) Transf. Leadership	413	3.32	0.92	0.12*	0.19**	0.21**	−0.13**	0.16**	0.06	0.20**	(0.82)						
(9) LMX	419	3.52	0.87	0.14**	0.10*	0.17**	−0.11*	0.18**	0.07	0.13**	0.85**	(0.74)					
(10) Proced. justice	417	2.81	0.73	0.28**	0.36**	0.42**	−0.26**	0.31**	0.15**	0.29**	0.23**	0.21**	(0.72)				
(11) Inform. justice	417	3.20	0.92	0.34**	0.33**	0.45**	−0.24**	0.30**	0.12*	0.22**	0.36**	0.31**	0.58**	(0.80)			
(12) Interp. justice	416	4.11	0.97	0.19**	0.00	0.15**	−0.13**	0.11*	0.08	0.09	0.69**	0.68**	0.19**	0.27**	(0.94)		
(13) Turnover intention	420	2.10	0.88	−0.14	0.00	−0.09	0.13**	0.03	−0.20**	−0.22**	−0.29**	−0.25**	−0.18**	−0.19**	−0.24**	(0.76)	
(14) Job satisfaction	420	3.85	0.83	0.20**	0.21**	0.32**	−0.19**	0.16**	0.16**	0.38**	0.49**	0.41**	0.29**	0.35**	0.30**	−0.56**	(0.76)

Reliabilities (Cronbach’s alpha) are indicated in the diagonal in brackets; **p < 0.05 (2-sided), *p < 0.1 (2-sided); *N,* sample size; M, mean value, SD, standard deviation.

**Figure 2:**
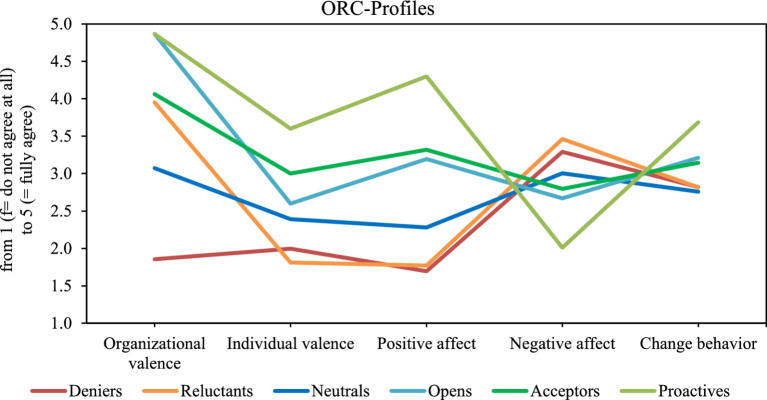
Typology of readiness for change.

Overall, the mean ORC value (=M) was 3.5 on a scale from 1 to 5, with a standard deviation (=SD) of 0.65. The 1-, 2-, 3-, 4-, 5-, 6-, and 7-class solutions were examined. To determine the best model fit, classes were added iteratively. The Akaike information criterion (AIC), Bayesian information criterion (BIC), and Sample size-adjusted BIC (SABIC) were used to select the most suitable model (see Table 2). Lower values of these criteria indicate a better solution. With each stepwise addition up to the 7-class model, all three values decreased, which can be seen in Table 1. However, since the 7-class solution had ≤2% of the sample assigned to a class which shows a reduced representability of the smallest group, the 6-class solution was chosen. An overview of the 6-class solution can be seen in Figure 2. The entropy was also higher in the models with more classes, indicating a better fit (Table 2).

**Table 2:** Results of LPA.

	2 profiles	3 profiles	4 profiles	5 profiles	6 profiles	7 profiles
Expected Parameters	16	22	28	34	40	46
LL	−2.628.948	−2574.138	−2541.925	−2.518.197	−2487.461	−2467.227
AIC	5.289.896	5.192.275	5.139.850	5.104.393	5054.922	5026.453
BIC	5.354.730	5.281.525	5.253.440	5.242.324	5217.194	5213.065
SABIC	5.303.956	5.221.710	5.164586	5.134.429	5090.259	5067.090
Entropy	0.845	0.784	0.799	0.789	0.817	0.845
aVLMR	0.0000	0.0000	0.3868	0.1057	0.3743	0.4785
BLRT	0.0000	0.0000	0.0000	0.0000	0.000	0.000

LL, loglikelihood; AIC, Akaike information criterion; BIC, Bayesian information criterion; SABIC, Sample size adjusted BIC; aVLMR, adjusted Vuong-Lo-Medell-Rubin test; BLRT, bootstrap likelihood ratio test.

The six identified classes can be described as follows: The first class was labeled the *Deniers*. *Deniers* rate the organizational and individual valence of the change process very low, experience little positive affect and high negative affect, while exhibiting comparatively low change behavior. The second class was labeled the *Reluctants*. While they rate the organizational valence to be high, they report low individual valence and positive affect, high negative affect and comparatively low change behavior, similar to the *Deniers*. The third class was labeled the *Neutrals*. The *Neutrals* are characterized by medium values in all facets but exhibits the lowest change behavior. The fourth class was labeled the *Opens*. This class shows very high organizational and medium individual valence. Positive and negative affect as well as change behavior are in the upper middle range. The fifth class was labeled the *Acceptors*. Those show slightly higher values in the facets of individual valence, positive and negative affect, similar values in change behavior, and lower values in organizational valence than the *Opens*. The sixth class was labeled the *Proactives*. *Proactives* have the highest overall ORC. They rate comparatively high in the facets of organizational valence, individual valence, positive affect, and change behavior, as well as rather low in negative affect. An overview of the descriptive values of the classes scored on each of the five facets of ORC can be seen in Table 3.

**Table 3:** Descriptive statistics of profile.

		Org. valence	Ind. valence	Pos. affect	Neg. aff. Inverted	Change behavior	Overall
Deniers(*N* = 15; 3.5%)	M	1.9	2.0	1.7	3.3	2.8	1.9
	SD	0.9	0.7	0.8	0.8	0.6	0.8
Reluctants(*N* = 34; 8.0%)	M	4.0	1.8	1.8	3.5	2.8	2.4
	SD	0.1	0.7	0.4	0.7	0.7	1.0
Neutrals(*N* = 31; 7.2%)	M	3.1	2.4	2.3	3	2.8	2.3
	SD	0.1	0.7	0.4	0.7	0.7	0.7
Opens(*N* = 97; 22.7%)	M	4.9	2.6	3.2	2.7	3.2	3.2
	SD	0.1	0.7	0.4	0.7	0.7	1.0
Acceptors(*N* = 104; 24.4%)	M	4.1	3.0	3.3	2.8	3.1	3.1
	SD	0.1	0.7	0.4	0.7	0.7	0.7
Proactives(*N* = 146; 34.2%)	M	4.9	3.6	4.3	2.0	3.7	3.9
	SD	0.1	0.7	0.4	0.7	0.7	0.7
Overall	M	3.8	2.6	2.8	1.9	3.1	–
	SD	1.2	0.7	1.0	0.7	0.4	–

M, Mean value; SD, Standard deviation; Org. valence, Organizational valence; Ind. valence, individual valence; Pos. affect, Positive affect; Neg. aff. inverted, Negative affect inverted; Overall, total mean value of the profile mean values with inverted values for negative affect and their SD.

A multinomial logistic regression was calculated to analyze predictors and outcome variables of the profiles, with *Proactives* chosen as the reference category. Results can be seen in Table 4 and descriptive statistics of predictors can be seen in Table 5. As assumed in Hypotheses 2b, 2f, and 2 g, employees who reported high values in optimism (*B* = −1.143; *p* = 0.000), interpersonal (*B* = −0.944; *p* = 0.042), and informational justice (*B* = −1.974; *p* = 0.004) were more likely to be assigned to the *Proactives* than to *Deniers*. Contrary to hypotheses 2a, 2c, 2d, and 2e, age (*B* = 0.045; *p* = 0.146), transformational leadership (*B* = 0.081; *p* = 0.925), LMX (*B* = 0.253; *p* = 0.754), and procedural justice (*B* = −1.025; *p* = 0.162) did not significantly predict membership in the *Proactives* profile compared to *Deniers*. Table 5 shows that the *Proactives* have the highest average values and the lowest variances in the predictors optimism (*M* = 6.0; SD = 0.1), transformational leadership (*M* = 3.6; SD = 0.1), LMX (*M* = 3.7; SD = 0.1), procedural (*M* = 3.3; SD = 0.1), interpersonal (*M* = 3.9; SD = 0.1), and informational justice (*M* = 3.9; SD = 1.1).

**Table 4:** Results of multinominal logistic regression (R3STEP) with profile *Proactives* as reference group.

Type		Age	Opt.	TL	LMX	Proc. j.	Interp. j.	Inf. j.
Deniers	B	0.045	−1.143	0.081	0.253	−1.025	−0.944	−1.974
	S.E.	0.031	0.307	0.858	0.807	0.732	0.463	0.694
	B/S.E.	1.454	−3.728	0.095	0.313	−1.400	−2.037	−2.845
	*p*	0.146	0.000*	0.925	0.754	0.162	0.042*	0.004*
Reluctants	B	0.031	−0.653	0.331	−0.199	−1.635	−0.423	−0.999
	S.E.	0.025	0.264	0.577	0.573	0.540	0.369	0.378
	B/S.E.	1.252	−2.469	0.573	−0.348	−3.027	−1.146	−2.64
	*p*	0.211	0.014*	0.567	0.728	0.002*	0.252	0.008*
Neutrals	B	0.016	−0.533	0.037	0.006	−1.199	−0.394	−0.348
	S.E.	0.019	0.204	0.448	0.452	0.424	0.305	0.319
	B/S.E.	0.836	−2.612	0.083	0.013	−2.831	−1.293	−1.090
	*p*	0.403	0.009*	0.934	0.989	0.005*	0.196	0.276
Opens	B	0.032	−0.606	−0.763	0.631	−1.237	0.036	−0.555
	S.E.	0.021	0.247	0.564	0.468	0.578	0.427	0.504
	B/S.E.	1.496	−2.449	−1.352	1.348	−2.141	0.085	−1.101
	*p*	0.135	0.014*	0.176	0.178	0.032*	0.933	0.271
Acceptors	B	0.019	−0.862	−0.996	1.288	−1.852	−0.571	−1.14
	S.E.	0.032	0.246	0.755	0.728	0.609	0.461	0.534
	B/S.E.	0.613	−3.500	−1.319	1.770	−3.042	−1.237	−2.135
	*p*	0.540	0.000*	0.187	0.077**	0.002*	0.216	0.033*

Opt., Optimism; TL, Transformational leadership; LMX, Leader member exchange; Proc. j., Procedural justice; Interp.j., Interpersonal justice; Inf. j., Informational justice; B, Regression coefficient; S.E., Standard error; B/S.E., z-value; *p ≤ 0.05.

**Table 5:** Descriptive statistics of predictors.

		Age	Opt.	TL	LMX	Proc. j.	Interp. j.	Inf. j.
Deniers	M	40.6	4.3	2.8	3.1	2.2	3.2	2.1
	SD	2.6	0.4	0.3	0.3	0.2	0.4	0.2
Reluctants	M	40.2	4.5	3.1	3.4	2.2	3.9	2.5
	SD	2.3	0.3	0.2	0.2	0.2	0.2	0.2
Neutrals	M	41.1	5.0	3.2	3.3	2.5	3.9	2.7
	SD	1.9	0.3	0.2	0.2	0.1	0.2	0.1
Opens	M	42.4	4.9	3.1	3.5	2.6	4.8	2.9
	SD	1.4	0.2	0.2	0.2	0.1	0.1	0.1
Acceptors	M	39.8	5.3	3.3	3.4	2.8	3.9	3.2
	SD	1.4	0.1	0.1	0.1	0.1	0.1	0.1
Proactives	M	40.9	6.0	3.6	3.7	3.3	3.9	3.9
	SD	1.1	0.1	0.1	0.1	0.1	0.1	0.1

Opt., Optimism; TL, Transformational leadership; LMX, Leader member exchange; Proc. j., Procedural justice; Interp.j., Interpersonal justice; Inf. j., Informational justice. M, Mean value; SD, Standard deviation.

Further, Table 4 shows that optimism predicts membership in the *Proactives* group compared to all other types (*p* ≤ 0.05). With the exception of the *Deniers*, procedural justice also predicts whether a person is more likely to belong to the *Proactives* or another type (*p* ≤ 0.05). Additionally, informational justice distinguishes whether an individual is part of the *Reluctants* or *Proactives*.

Furthermore, it was shown that the mean values of job satisfaction (χ^2^ = 71. 627, *p* < 0.001) and intention to leave (χ^2^ = 33.359, *p* < 0.001) significantly differ between the profiles, supporting hypotheses 3a and 3b (see Table 6). Post-hoc tests with Bonferroni correction were calculated. No significant differences were found in these pairwise comparisons (*p* ≥ 0.05). It should be noted that the χ^2^ test focuses on the overall differences between the groups, while the post-hoc tests examine specific pairwise comparisons between two groups (Cabin and Mitchell, 2000; Dunkl et al., 1990; Franke et al., 2012). The significant χ^2^ test indicates differences between the groups, but these are not considered significant in pairwise comparisons.

**Table 6:** Results of LPA with distal outcomes (DU3STEP).

		Job satisfaction	Intention to leave
Deniers	M	3.1	3.0
	SD	0.3	0.3
Reluctants	M	3.3	0.2
	SD	0.2	0.2
Neutrals	M	3.7	2.1
	SD	0.1	0.1
Opens	M	3.6	2.4
	SD	0.1	0.1
Acceptors	M	3.8	2.2
	SD	0.1	0.1
Proactives	M	4.3	1.7
	SD	0.1	0.1
Overall	χ^2^	71.6	33.4
	*p*	< 0.001	< 0.001

## 5 Discussion

The aim of this study was to develop a comprehensive typology of ORC that encompasses the three levels of cognitive, affective and behavioral ORC in organizations. Additionally, the study sought to identify antecedents and consequences of profile membership. An ORC typology is presented, based on the five factors of the Organizational Readiness for Change Questionnaire (Gräfe and Kauffeld, 2023), using latent profile analysis. The six identified types are labeled *Deniers*, *Reluctants*, *Neutrals*, *Opens*, *Acceptors* and *Proactives*. The greatest variances between the mean values of the groups were observed in organizational valence and positive affect, indicating the most significant differences among the six types. The *Deniers* descriptively displayed the lowest levels of both organizational valence and positive affect, whereas the *Proactives* and *Opens* descriptively reported the highest organizational valence, with the *Proactives* also descriptively exhibiting the strongest positive affect. Moreover, the *Proactives* descriptively demonstrated the least negative affect toward change and descriptively perceived the highest individual valence regarding the change. This suggests that the *Proactives* recognize the greatest benefits from change for both the organization and themselves, and they have a highly positive emotional response to it. Additionally, they descriptively exhibit the strongest change behavior, which, according to the used questionnaire, indicates they are above average in seeking information, exchanging ideas with colleagues, and articulating their opinions about the change (Maier et al., 2007). In contrast, the *Deniers* descriptively engage in these behaviors significantly less frequently, perceive descriptively little valence of the change for either the organization or themselves, and exhibit descriptively low positive affect along with above-average negative affect toward the change. As previously mentioned, *Opens*, alongside *Proactives*, descriptively possess the highest organizational valence. However, they descriptively perceive the relevance of change for themselves as significantly lower than *Proactives* do. This difference may lead to a weaker positive affect, a stronger negative affect, and reduced change behavior among *Opens*. Thus, it can be hypothesized that individual valence may be particularly crucial for typological affiliation. However, these results rely on descriptive differences only and should be tested in detail in future research.

The study shows that optimism as an individual attribute, along with the two contextual variables interpersonal and informational justice, plays a critical role in determining whether an individual is more likely to belong to the *Proactives* compared to the *Deniers*. Furthermore, regarding the lower levels of informational justice among the *Deniers*, it is plausible that they lack essential information, which contributes to their unawareness of the relevance of the change. This aligns with the finding that they perceive informational justice as the lowest among all types. It could be assumed that these individuals may be employees with limited access to informational resources, such as lacking a PC or laptop to access the company’s intranet. This suggestion warrants further investigation. It is also shown that procedural justice is not a predictor of whether someone is more likely to be a *Proactive* or a *Rejector*, but instead allows discrimination between *Proactives* and all other types. Transformational leadership, LMX, and procedural justice as well as the age are no predictors for type membership. It is also demonstrated that job satisfaction and the intention to leave can be predicted based on profile membership.

The types exhibit the greatest differences in organizational valence and positive affect, while they show the least variation in negative affect and change behavior. The minimal difference in change behavior may be attributed to limited opportunities for employees to demonstrate change behavior. Employees might not have sufficient time to engage deeply in discussions about the change, yet they likely still have some opportunities for exchange through shift discussions and similar interactions. Additionally, it is noteworthy that the *Deniers* and *Reluctants* display nearly identical characteristics of ORC, with the exception of organizational valence, suggesting that cognition may not be directly linked to the affect and change behavior of the types. It remains unclear whether an increase in organizational valence would lead a *Rejector* to become a *Resistor*, or if such a change might also correspond with a transition to, for example, a *Neutrals* type, which would likely be accompanied by higher values on the other scales.

### 5.1 Theoretical implications

This study reveals various theoretical implications, advancing a typology that extends beyond previous findings. A key result is that employees respond to change in varying ways which is conform with previous theories (e.g., Oreg et al., 2018; Gräfe and Kauffeld, 2023). As noted, the most significant variances in type mean values were found in organizational valence and positive affect, indicating that types differ markedly in these areas. This could suggest that organizational valence and positive affect are strong predictors of the overall mean value of ORC implying that higher positive affect correlates with higher ORC. However, our results contradict this assumption; for example, *Neutrals* have a higher positive affect than *Reluctants*, yet *Reluctants* display a higher overall ORC than *Neutrals*. This finding challenges the conclusions drawn by, who based their typology exclusively on affect to infer behavioral outcomes. Thus, the typology presented here emphasizes that ORC should be viewed as a multidimensional construct (Rafferty et al., 2013).

The developed typology shares some similarities and differences with previous models. In the Circumplex of Change Recipients’ Responses to Change and Underlying Core Affect by Oreg et al. (2018), the focus is on affect, with the dimensions of activation (representing the degree of pleasantness or positivity) and valence (indicating the level of arousal). In our study, we used positive and negative affect besides the other facets of ORC to build the typology (Gräfe and Kauffeld, 2023). These dimensions reflect both valence, for example, when more positive than negative affect is present, and activation, by examining the intensity of the affective responses. So, it can be assumed that Oreg et al. (2018) “Change Proactivity” type in our study would be characterized by a high level of positive affect and a low level of negative affect. Accordingly, we named our type with these characteristics *Proactives*, in line with Oreg et al. (2018). In their study, no specific emotions were suggested for each type, but it is likely that the emotions associated with the “Change Proactivity” class (e.g., excited, elated, enthusiastic) proposed by Oreg et al. (2018) could also apply to the *Proactives*. The Circumplex of Change Recipients’ Responses to Change does not consider shared emotions which is a limitation that the authors themselves acknowledge (Oreg et al., 2018). Based on the findings from our study on the facets of ORC, we assume that a type can experience both positive and negative affect simultaneously (Gräfe and Kauffeld, 2023). Our results show that all types demonstrate both positive and negative affect, revealing notable limitations in Oreg et al. (2018) typology. Furthermore, Oreg et al. (2018) focuses exclusively on affect, making assumptions about the other two facets cognition and behavior. Our findings suggest that inferring one facet’s expression based on the others is not straightforward; the relationship between cognition, affect, and behavior is significantly more complex. This complexity aligns with insights from researchers such as Piderit (2000), emphasizing the need for a more comprehensive examination of these facets.

The Transtheoretical Model by Prochaska and DiClemente (1992) primarily addresses behavioral change. Its primary focus is on changing behavior, whereas our typology emphasizes readiness for change, which encompasses not only behavior but also cognition and affect. While our types differ in the degree of change behavior, it became evident that they vary more strongly in the other facets of ORC. So, behavior itself seems to play a minor role when distinguishing ORC types. The first phase of the Transtheoretical Model, Contemplation, is characterized by individuals being unaware of the existence of a problem (Prochaska and DiClemente, 1992). Applied to our typology, this could suggest that their valence is very low because they are unaware of the need for change which characterizes the *Rejectors*. In the Action stage of the Transtheoretical Model, people actively modify their behavior, which could be comparable to our *Proactives*, who demonstrate the strongest change behavior. Another distinction lies in the fact that the phases in the Transtheoretical Model refer to specific phases, whereas our typology applies to specific individuals. This difference underscores that the Transtheoretical Model focuses on the temporal progression through stages of change a person undergoes, while we focus on personal tendencies. Given the existing parallels between the phases of the Transtheoretical Model and the ORC types, it is reasonable to assume that these types may also evolve over time (Prochaska and DiClemente, 1992).

Furthermore, the study sheds light on the factors associated with this typology, illustrating their influence on key organizational outcomes, such as intention to leave and job satisfaction. This underscores the critical importance of ORC research and reinforces previous findings (Wanberg and Banas, 2000). It highlights the necessity of considering ORC profiles to better understand job satisfaction and intentions to leave during organizational changes (Wanberg and Banas, 2000). The identified influencing factors, such as optimism and both interpersonal and informational justice, align with existing research (Armenakis et al., 2007; Avey et al., 2011; Gräfe and Kauffeld, 2023). Additionally, while the observed trends regarding LMX, transformational leadership and procedural justice resonate with prior findings, the lack of significant results suggests their limited impact on type membership in contrast to previous research, which relates the influence of these predictors to the ORC (Armenakis et al., 2007; Banguntopo, 2018; Bernerth et al., 2007; Van Dam et al., 2008). The fact that transformational leadership and LMX did not significantly predict membership in the *Proactives* profile compared to the *Deniers* may be attributed to a multifaceted interplay of individual, team-level, cultural, and contextual factors. For transformational leadership and LMX, personality traits such as agreeableness and conscientiousness may play a homogenizing role, as employees with these traits tend to rate their leaders positively regardless of their profile (Bauer and Green, 1996). Team dynamics could further diminish the influence of these leadership factors, as employees’ behaviors and attitudes are often shaped more strongly by team cohesion and collective norms than by individual leadership relationships or inspirational leadership elements (Sparrowe and Liden, 1997; Seers, 1989). In organizations with strong cultural norms, collective guidelines may overshadow individual leadership styles, making behaviors more aligned with institutional expectations than with dyadic relationships (Chatman and O’Reilly, 2016). Similarly, situational demands in dynamic or project-based environments often require employees to focus on task-specific adaptations rather than on leadership relationships (Yukl and Mahsud, 2010). Nevertheless, previous studies showed significant influences of LMX and transformational leadership on ORC (e.g., Gräfe and Kauffeld, 2023). So, the lack of a significant effects on ORC profiles could further be attributed to the loss of information inherent in the creation of these profiles. Reducing continuous data on ORC into discrete profiles eliminates a portion of the variance that might otherwise explain the relationship between leadership and readiness for change. This process results in potentially relevant individual differences within the profiles being disregarded, which can lead to an underestimation of the strength of the association. Additionally, profile formation reduces statistical power, especially when participant distribution across profiles is uneven or sample sizes within profiles are small. Reduced statistical power makes it more challenging to reliably detect existing effects, even when they are present (Cohen, 1992). For age, the absence of a significant or descriptive trend reflects broader inconsistencies in the literature regarding its role in shaping employees’ openness to change. Some studies suggest younger employees are more adaptable due to greater flexibility, while others show no consistent relationship or even argue that older employees may engage more positively in transformational efforts under the right conditions (Ng and Feldman, 2013). Contextual factors, such as self-efficacy, prior experiences with change, and organizational emphasis on continuous learning, often play a more decisive role than age (Maurer, 2001). The lack of a statistically significant difference in procedural justice between the *Proactives* and the *Deniers*, despite significant differences with other profiles, can further be attributed to the small sample size of the Deniers group. Small sample sizes reduce statistical power, making it harder to detect significant effects. They also inflate the standard error, leading to less precise estimates and wider confidence intervals, which diminishes the ability to achieve statistical significance. Furthermore, smaller samples often dampen effect sizes, limiting the model’s capacity to distinguish true differences from random variability (Tabachnick et al., 2019; Field, 2018).

### 5.2 Practical implications

The ORC typology developed in this study can be applied in various practical ways. Organizations facing or undergoing change can use the typology to familiarize employees and leaders with the concept of ORC. It provides a framework for intuitively understanding employee reactions and can be used to encourage self-reflection among employees. Leaders can also use the typology to classify employees’ behaviors and guide their support accordingly. Additionally, organizations can empirically assess ORC profiles to gain insights into employee perceptions of change. This can be done at the department or organizational level to ensure anonymity and provide a broader understanding of how different groups view the change process. Such insights can inform interventions, helping to address potential areas of concern and supporting succession planning by predicting retention risks.

Organizations should prioritize strengthening the dimensions of ORC across all employees, with particular attention to those categorized as *Deniers*, *Reluctants* and *Neutrals*. Enhancing organizational valence can be achieved through clear and transparent communication, ensuring that employees comprehend the underlying rationale and necessity of the change process. In instances where the change is unavoidable, it is essential to communicate this from the outset, providing clarity and reducing uncertainty. To improve individual valence, it is crucial to highlight the personal benefits of the change, enabling employees to perceive how it might positively impact their roles and professional growth. Additionally, organizations may consider implementing incentive systems, such as performance-based bonuses, to further motivate employees and increase their engagement with the change. Furthermore, organizations should ensure that sufficient opportunities are provided for employees to demonstrate change behavior. This entails giving employees access to all pertinent information, encouraging active engagement with colleagues, and fostering an environment where they can openly express their views. Initiatives such as facilitated discussion sessions or workshops can be instrumental in enabling employees to share their perspectives and contribute their own ideas. Moreover, the introduction of an anonymous feedback mechanisms could serve as a valuable tool, allowing employees to provide candid feedback on the change process without concern for personal repercussions. In addition, it is imperative to cultivate positive emotional responses toward the change. Leadership might play a pivotal role in this regard, as leaders should convey information about the change with confidence, enthusiasm, and a positive outlook. This approach can help generate a sense of collective purpose and shared optimism within the organization. Creating a culture of “team spirit” or fostering a sense of renewal can further reinforce these positive emotions. At the same time, efforts should be made to minimize negative affect by reducing any perceived disadvantages that the change might pose to employees, thus making the change process more appealing and acceptable.

The typology can also be integrated into personnel development measures. The ORC typology can be a practical instrument for Human Resources. Nevertheless, it must be applied carefully to avoid possible discrimination by, e.g., avoiding hiring certain types to prevent problems in ORC. Furthermore, *Proactives* can be trained to serve as internal “change champions,” using their influence to promote the benefits of change to their peers. Their credibility and trust among colleagues make them effective communicators, capable of inspiring others to embrace the change. Engaging groups like “Opens” and “Acceptors” early can help these employees transition into *Proactives*, further expanding the base of support for change. For employees with less ORC like *Deniers*, *Reluctants*, and *Neutrals*, targeted development measures are essential. This study highlights optimism, interpersonal and informational justice as key factors in increasing readiness. Companies can promote resilience through interventions such as mindfulness training, which has been shown to improve optimism. Companies could, for example, create measures for employees that strengthen their optimism, a component of resilience. Thus, it might be useful to strengthen overall resilience as psychological resistance in stressful situations like change processes (Henninger, 2016). One study shows that, for instance, web-based mindfulness training strengthens the resilience of employees (Pauls et al., 2016). Ensuring transparent and regular communication fosters informational justice, while open, respectful interactions between managers and employees reinforce interpersonal justice. Informational justice is crucial for ORC type membership. Employees who feel underinformed or excluded from the decision-making process are more likely to be a *Rejector* than others. Organizations should regularly provide truthful, specific, and timely information about the change to promote informational justice, helping all employees feel informed and engaged (Colquitt et al., 2023; Maier et al., 2007). Another factor influencing type membership is interpersonal justice. Awareness-raising measures could be useful here to emphasize the relevance of mutual harmony. Also, interactions between companies or managers and employees should be appropriate measures to strengthen interpersonal justice. Especially for types *Deniers, Reluctants* and *Neutrals*, Motivational Interviewing can be particularly effective (Klonek and Kauffeld, 2012; Rollnick and Miller, 1995). This is an approach to communication by strengthen a person’s motivation and commitment to change to strengthen change behavior (Endrejat and Kauffeld, 2021; Güntner et al., 2021; Rollnick and Miller, 1995). Originally from clinical psychology, this approach can be successfully used in companies by building intrinsic motivation for behavioral change through targeted communication (Güntner et al., 2021). Due to the fact, that the perceived transformational leadership, LMX and procedural justice are descriptively higher for Proactives than for the other types, it could be additionally helpful, to improve these. Even though the results are not significant, it can be assumed that this will have a positive rather than a negative effect on the ORC like previous studies showed (Armenakis et al., 2007; Banguntopo, 2018; Bernerth et al., 2007; Van Dam et al., 2008). In conclusion, the ORC typology provides a structured framework for navigating organizational change. By addressing the specific needs of different employee groups, ensuring clear communication, and regularly assessing readiness, organizations can cultivate a more resilient and adaptable workforce. Overall, it should be noted that both organizations and leaders can contribute to the ORC types to which employees belong to.

### 5.3 Limitations and future research

While the developed typology has many interesting theoretical and practical implications, there are some restrictions that need to be taken into account. First, the 6-class solution was selected even though the fit indices AIC, BIC, and SABIC, as well as entropy, favored the 7-class solution. This solution might have provided an even better fit but was not chosen ultimately because the small group size of the seventh group (less than 2%). This paper argues that the 6-class solution offers a sufficiently differentiated view of individual reactions to change processes, while offering meaningful classes and not overfitting the data. Secondly, the sample is not evenly distributed across the six classes, which means that types with lower ORC are less strongly represented. This uneven distribution can be attributed to the fact that the employees in the sample tend to be more positive about change, which is why the types with lower readiness for change may occur less frequently. This limitation can therefore be attributed to the sample under consideration and invalidated. Nevertheless, the unequal group sizes, particularly the small size of the *Deniers* group, may have led to reduced statistical power, resulting in higher standard errors and less precise estimates. This could explain why e. g. the difference in procedural justice between the *Deniers* and the *Proactives* did not reach statistical significance, despite observable descriptive trends. Third, it is noteworthy that 98% of the respondents were male, reflecting the high proportion of men in the industry of the considered sample. Research indicates that women might be more open to change than men (McManus et al., 2008), so that including more women might have identified further profiles with high ORC. For sectors in which the proportion of women is higher, it can be assumed that the suggested types can still be found. The imbalanced gender distribution may have influenced the results regarding the perception of organizational change and justice. Research indicates that men and women often respond differently to workplace changes. For instance, Ely and Meyerson (2000) found that men are more likely to adapt to traditional power structures, while women tend to favor more communicative and inclusive approaches to change. Additionally, women generally are more sensitive to interpersonal and informational injustices, which might not be fully captured in my predominantly male sample (Lee and Farh, 1999). As a result, the findings on informational justice and its role in understanding organizational change may be skewed by the gender imbalance. To make more generalizable claims, it would be necessary either to use a more balanced sample or to critically consider the results in light of this gender distribution. It is recommended to expand future research to include other sectors in order to ensure the generalizability of the ORC typology. Specifically, incorporating companies from different countries and those with a higher proportion of women could reveal gender-specific differences in openness to change. This may also lead to the identification of new typologies that were not present in the current sample. Fourth, all data were collected from the same source (self-assessment), which could pose a challenge to the interpretation of the results due to potential data bias. Future studies should therefore collect external estimations (e.g., from colleagues or supervisors). However, this limitation is mitigated by the use of validated questionnaires for all assessed variables. Fifth, the study participants are employees of the same company. This could be seen as a limitation since the sample was not fully random and generalizability might be restricted. Nevertheless, six types could be found that differ in affect, cognition and behavior. Lastly, the results are based on a cross-sectional design. Therefore, the development of various variables of ORC types over time cannot be analyzed. It is expected, however, that the membership in one of the identified classes could change over time due to various influencing factors. Therefore, an important next step in research is to observe the types in a longitudinal design to determine the stability of the profiles and to identify what it depends on if the ORC profiles change over time.

Future research should further aim to replicate and extend the findings regarding transformational leadership, LMX, procedural justice and age to better understand their roles in shaping profile membership. For age, future studies could explore whether its lack of significance in this study reflects a genuine absence of effect or whether it is due to the contextual or sample-specific nature of the data. Given the inconsistent findings in the literature about the relationship between age and ORC, further research should investigate whether age-related differences emerge in other organizational settings or under varying circumstances. Additionally, larger and more evenly distributed samples across age groups could clarify whether the trends observed in this study are the result of statistical limitations or if age truly has a limited impact on employee profiles. For procedural justice, further research could examine whether equal group sizes lead to significant between *Deniers* and *Proactives*. Additionally, larger and more evenly distributed samples would help clarify whether the observed trends are reflective of genuine effects or a result of statistical limitations. By addressing these aspects, future studies could provide more conclusive evidence regarding the significance and practical implications of these variables. Addressing these aspects would provide a more comprehensive understanding of how demographic and leadership variables contribute to profile differentiation.

Further, the factor content, which represents the fourth factor in the model by Holt et al. (2007) alongside individual attributes, context and process variables, was not examined as an influencing factor on the typology, as all employees in this sample undergo the same change process. It is therefore advisable to apply and test the typology using other change projects. It is also conceivable to assess employees in terms of their ORC type with regard to various change projects. This could allow a statement to be made about the extent to which employees of one type always react to different changes in similar patterns or not. Moreover, following Oreg et al. (2018), it could be useful to assign different emotions to the types to achieve a better understanding. Further, it can be assumed that profile memberships may change over time depending on factors such as justice, as e.g., the organizational valence could increase by giving employee more transparent and fair information which can lead to another type membership over time. This assumption also underlies the Transtheoretical Model (Prochaska and DiClemente, 1992). If a company, for example, finds that many employees belong to the *Deniers* at the beginning of a change and then develops a change strategy incorporating an early, effective information and communication culture, it is expected that a further measurement of profiles would show significantly fewer individuals belonging to the *Deniers*. Conversely, it must also be assumed that a deterioration in profiles is possible, so that the *Proactives* could evolve into *Acceptors*, for example, due to very low perceived justice. Based on this, it is important for further research to consider the dynamics of the types over time and also to determine other influencing factors and consequences. It may also be possible to identify mediating or moderating variables that could further increase complexity. Overall, the effects on type membership and the consequences of this should be investigated in more detail in further research.

## 6 Conclusion

In summary, this paper revealed six types of employees’ ORC which differ in affect, cognition and behavior named *Deniers*, *Reluctants*, *Neutrals*, *Opens* and *Acceptors*. This typology expands on previous research, where typologies have been built, but without a holistic view of the construct. It helps to reduce the complexity of ORC for an easy practical usage. It was shown that employees with strong optimism and high perceived interpersonal and informational justice are more likely to belong to the *Proactives* than to the *Deniers* whereas age, transformational leadership, LMX do not significantly influence this. In addition, job satisfaction and turnover intention are significant consequences of type. The findings can be used in practice as starting points for understanding and strengthening employees’ ORC. Future research should focus testing the ORC typology in several sectors in different countries with a mixture of female and male employees to ensure a high representatively.

## Data Availability

The raw data supporting the conclusions of this article will be made available by the authors, without undue reservation.

## Appendix

The following table shows the used measurements. The survey was conducted using the original German items. For the scales on transformational leadership, LMX, proceural justice, interpersonal justice, informational justice and job satisfaction we cited the original English items for better understanding of readers (Bass and Avolio, 1995; Graen and Uhl-Bien, 1995; Colquitt, 2001). For the other used scales we used backtranslation method to translate the used original German items into English for better understanding of this manuscript.

Measurements with example items.

**Table:**

Construct	Measurement	Example item original in German	Example item in English
Organizational Readiness for Change	Organizational Readiness for Change Questionnaire (Gräfe and Kauffeld, 2023)	“Die Veränderung ist wichtig für unsere Organisation.”	“The change is important for our company.”
Optimism	SOP2 (Kemper et al., 2013)	“Optimisten sind Menschen, die voller Zweifel in die Zukunft blicken und meistens Schlechtes erwarten. Bitte schätzen Sie sich selbst ein: Wie optimistisch sind Sie im Allgemeinen?”	“Optimists are people who look to the future full of doubt and mostly expect bad things. Please rate yourself: How optimistic are you in general?”
Transformational Leadership	German validated version of the MLQ-5 x Short (Felfe, 2006)	“Spricht optimistisch über die Zukunft.”	“Talks optimistically about the future.” (Bass and Avolio, 1995)
Leader Member Exchange	German leader member exchange scale (Schyns, 2002).	“Wie gut versteht Ihre Führungskraft Ihre beruflichen Probleme und Bedürfnisse?”	“How well does your leader understand your job problems and needs?” (Graen and Uhl-Bien, 1995)
Procedural justice	German version of Measurement of Organizational Justice by Colquitt (2001) (Maier et al., 2007)	“Wie sehr konnten Sie Ihre Sichtweisen und Empfindungen während des Vorgehens ausdrücken?”	“Have you been able to express your views and feelings during those procedures?” (Colquitt, 2001)
Interpersonal justice	German version of Measurement of Organizational Justice by Colquitt (2001) (Maier et al., 2007)	“Wie sehr hat (er/sie) Sie höflich behandelt?”	“Has (he/she) treated you in a polite manner?” (Colquitt, 2001)
Informational justice	German version of Measurement of Organizational Justice by Colquitt (2001) (Maier et al., 2007)	“Wie sehr hat (er/sie) Ihnen Einzelheiten rechtzeitig mitgeteilt?”	“Has (he/she) communicated details in a timely manner?” (Colquitt, 2001)
Job satisfaction	Modified version of the Job Diagnostic Survey (Kil et al., 2000)	“Ich bin zufrieden mit meiner Arbeit.”	“I am satisfied with my work.” (Hackman and Oldham, 1975)
Intention to leave	Questionnaire by Baillod and Semmer (1994)	“Wie oft denken Sie selbst daran, Ihre Stelle zu verlassen?”	“How often do you think about leaving your job?”
